# Spatial and temporal trends of visceral leishmaniasis by mesoregion in a southeastern state of Brazil, 2002-2013

**DOI:** 10.1371/journal.pntd.0005950

**Published:** 2017-10-06

**Authors:** Thais Almeida Marques da Silva, Wendel Coura-Vital, David Soeiro Barbosa, Carla Sayuri Fogaça Oiko, Maria Helena Franco Morais, Bruna Dias Tourinho, Diogo Portella Ornelas de Melo, Ilka Afonso Reis, Mariângela Carneiro

**Affiliations:** 1 Laboratório de Epidemiologia das Doenças Infecciosas e Parasitárias, Departamento de Parasitologia, Instituto de Ciências Biológicas, Universidade Federal de Minas Gerais, Belo Horizonte, Minas Gerais, Brazil; 2 Instituto de Ensino e Pesquisa da Santa Casa Belo Horizonte, Belo Horizonte, Minas Gerais, Brazil; 3 Laboratório de Epidemiologia e Citologia, Escola de Farmácia, Universidade Federal de Ouro Preto, Ouro Preto, Minas Gerais, Brazil; 4 Secretaria Municipal de Saúde de Belo Horizonte, Belo Horizonte, Minas Gerais, Brazil; 5 Secretaria Estadual de Saúde de Minas Gerais, Minas Gerais, Brazil; 6 Departamento de Estatística, Instituto de Ciências Exatas, Universidade Federal de Minas Gerais, Belo Horizonte, Minas Gerais, Brazil; 7 Pós-graduação em Ciências da Saúde: Infectologia e Medicina Tropical, Faculdade de Medicina, Universidade Federal de Minas Gerais, Belo Horizonte, Minas Gerais, Brazil; The Faculty of Medicine, The Hebrew University of Jerusalem, ISRAEL

## Abstract

**Background:**

Visceral leishmaniasis (VL) is expanding in Brazil and in other South American countries, a process that has been associated with the urbanization of the disease. This study analyzes the spatial and temporal distribution of VL in the Brazilian state of Minas Gerais and identifies the areas with higher risks of transmission.

**Methodology:**

An ecological study with spatial and time series analyzes of new confirmed cases of VL notified to the Brazilian Notifiable Disease Information System between 2002 and 2013, considering the 12 mesoregions of Minas Gerais. Two complementary methodologies were used: thematic maps of incidence and Poisson (log-linear) generalized linear model. Thematic maps using crude and smoothed cumulative incidences were generated for four trienniums. Poisson Regression measured the variation of the average number of cases from one year to the following, for each mesoregion.

**Principal findings:**

The 5,778 cases analyzed revealed a heterogeneous spatial and temporal distribution of VL in Minas Gerais. Six mesoregions (Central Mineira, Jequitinhonha, Metropolitan area of Belo Horizonte, Northwest of Minas, North of Minas, and Vale do Rio Doce) were responsible for the expansion and maintenance of VL, with incidence rates as high as 26/100,000 inhabitants. The Vale do Rio Doce and Jequitinhonha mesoregions showed a considerable increase in the incidence rates in the last period studied. The other six mesoregions reported only sporadic cases and presented low and unsteady incidence rates, reaching a maximum of 1.2/100,000 inhabitants.

**Conclusions/Significance:**

The results contribute to further the current understanding about the expansion of VL in Minas Gerais and may help guide actions for disease control.

## Author summary

This article presents the spatial and temporal distribution of visceral leishmaniasis (VL) in Minas Gerais State and identifies the greater risk areas of transmission. This study is both timely and substantive because Minas Gerais is an important Brazilian state in the number of cases of visceral leishmaniasis. The results showed that during the 12-year time series the VL had a heterogeneous spatial and temporal distribution in the state of Minas Gerais. Among the 12 existing mesoregions, six (Central Mineira, Jequitinhonha, Metropolitan area of Belo Horizonte, Northwest of Minas, North of Minas, and Vale do Rio Doce) were responsible for the expansion and maintenance of VL in the state. Among them, the Vale do Rio Doce and Jequitinhonha mesoregions presented a considerable increase in the incidence rates of the disease in the last period. In the other six mesoregions only sporadic cases of the disease were reported during the study period. The results of in this study may contribute to a better understanding the dynamic of the disease in Minas Gerais. Also these findings can provide subsidies to assist the actions of the control program of VL.

## Introduction

Until 1980, visceral leishmaniasis (VL) was considered a strictly rural disease in Brazil, the main parasite (*Leishmania infantum*) reservoirs being foxes (*Dusicyon vetulus* and *Cerdocyon thous*) and marsupials (*Didelphis albiventris*) [1]. However, the epidemiological profile of VL shifted with the urbanization of the disease and domestic dogs became the main reservoir (*Canis familiaris*) [1,2]. Since the first VL epidemic in 1981 in Teresina, the capital of the state of Piauí located in northeastern Brazil, various epidemics occurred in other major urban centers in the northeast (São Luís, Natal, and Aracajú), north (Boa Vista and Santarém), mid-west (Cuiabá and Campo Grande), and southeast (Belo Horizonte and Montes Claros) regions of the country [3].

The geographic expansion of VL is associated with the process of urbanization of the disease. Indeed, migration of people from rural endemic areas to urban centers, adaptation of the vector to the domestic environment, the presence of disease reservoirs such as domestic dogs, malnutrition, and the lack of basic sanitation are considered contributing factors to the urbanization and geographic expansion of VL [3–5]. In this context, the Visceral Leishmaniasis Control and Surveillance Program (VLCSP) was implemented in Brazil to reduce the risk of transmission, the lethality, and the morbidity rates of VL in urban and rural areas. The program has three main pillars: the treatment of human cases, control of canine reservoirs, and vector control [1].

Aiming to identify areas where VLCSP strategies should be prioritized, a temporal study (2001–2011) of the disease’s incidence was conducted and pointed out the southeastern Brazilian state of Minas Gerais as a priority [6]. The VL incidence rates in this state were 1.6 and 1.4 for 100,000 inhabitants in 2012 and 2013, respectively. These rates were superior to those found for the entire southeast region (0.6 e 0.5/100,000 inhabitants) and similar to the national rates (1.6/100,000 inhabitants) [7].

The first cases of human VL in Minas Gerais were registered from 1940 in the North of Minas mesoregion [8] and, in the 1960’s, in the Vale do Rio Doce mesoregion [9]. In 1989, the first autochthonous case in an urban area was registered in the municipality of Sabará [10], which belongs to the metropolitan region of Belo Horizonte. Later, in 1994, the first autochthonous case was registered in Belo Horizonte, the capital of the state [11].

Currently, some cities of the state of Minas Gerais are considered endemic for VL and have attracted studies regarding the disease. Among those, stands out Montes Claros [12] and Porteirinha [13] in the North of Minas mesoregion; Paracatu [14] in the Northwest of Minas mesoregion; and Belo Horizonte in the Metropolitan area of Belo Horizonte mesoregion [15,16]. Governador Valadares, located in the Vale do Rio Doce mesoregion, registered cases of human VL in the 1960’s [9] and, following disease control measures, the municipality became a silent area for VL [17]. Unfortunately, disease control actions were interrupted in the 1990’s [18,19] and reporting of VL cases resumed in 2008 [19,20] to the point that, currently, Governador Valadares is considered a re-emergent focus of intense VL transmission [19].

Several studies have attempted to understand VL urbanization and geographic expansion by means of spatial and temporal analyses. They studied the distribution and variation of the incidence rates of human [6,21–23] the distribution of canine infection cases [24,25] the abundance of phlebotomine sand flies [25–27], and the temporal trends of the disease [23,28,29]. Others identified areas where VL control measures could be prioritized [30–32].

Studies conducted in other Brazilian states such as Pernambuco [21], Mato Grosso do Sul [22], São Paulo [33,34] and Maranhão [35] evaluated the dispersion of VL over time. To our knowledge, no study to date has evaluated the VL dispersion in the state of Minas Gerais and therefore more robust and updated research is needed to unveil the VL profile in this area. Indeed, a combination of different methodologies for spatial and temporal analyses of VL may be useful to understand the aggregation, maintenance, and dispersion patterns of the disease, not only in Minas Gerais, but also in other regions of Brazil.

The present study analyzed the spatial and temporal distribution of VL in the state of Minas Gerais between 2002 and 2013 using two different methodologies to further understand, characterize, and quantify the expansion of the disease in the vast territory occupied by this Brazilian state. The results of this study identify within the state the areas that could be prioritized by the control and vigilance of VL, considering the specificities of each mesoregion of the state of Minas Gerais.

## Methods

### Ethical statement

This study was approved by the Ethics Committee on Research of the Federal University of Minas Gerais (UFMG) under the number CAAE n.45497015.3.0000.5149. Only secondary information about the patients was collected from the Brazilian Notifiable Disease Information System (SINAN/VL). Data were analyzed anonymously.

### Design and area of study

This is an ecological study analyzing the spatial and temporal patterns of confirmed cases of VL notified to the SINAN/VL in the period between 2002 and 2013, in which the units of analysis were the 12 mesoregions of Minas Gerais.

Minas Gerais is one of the 27 federative units of Brazil, being located in the southeast region of the country. It has an area of 586,521.24 km² and ranks as the fourth largest state in territorial extension. With the highest number of municipalities (853) in the country, Minas Gerais is the second most populous Brazilian state with an estimated population of 20,997,560 in 2016, and a population density of 33.41 inhabitants/km^2^. The capital is Belo Horizonte [36].

**Fig 1:**
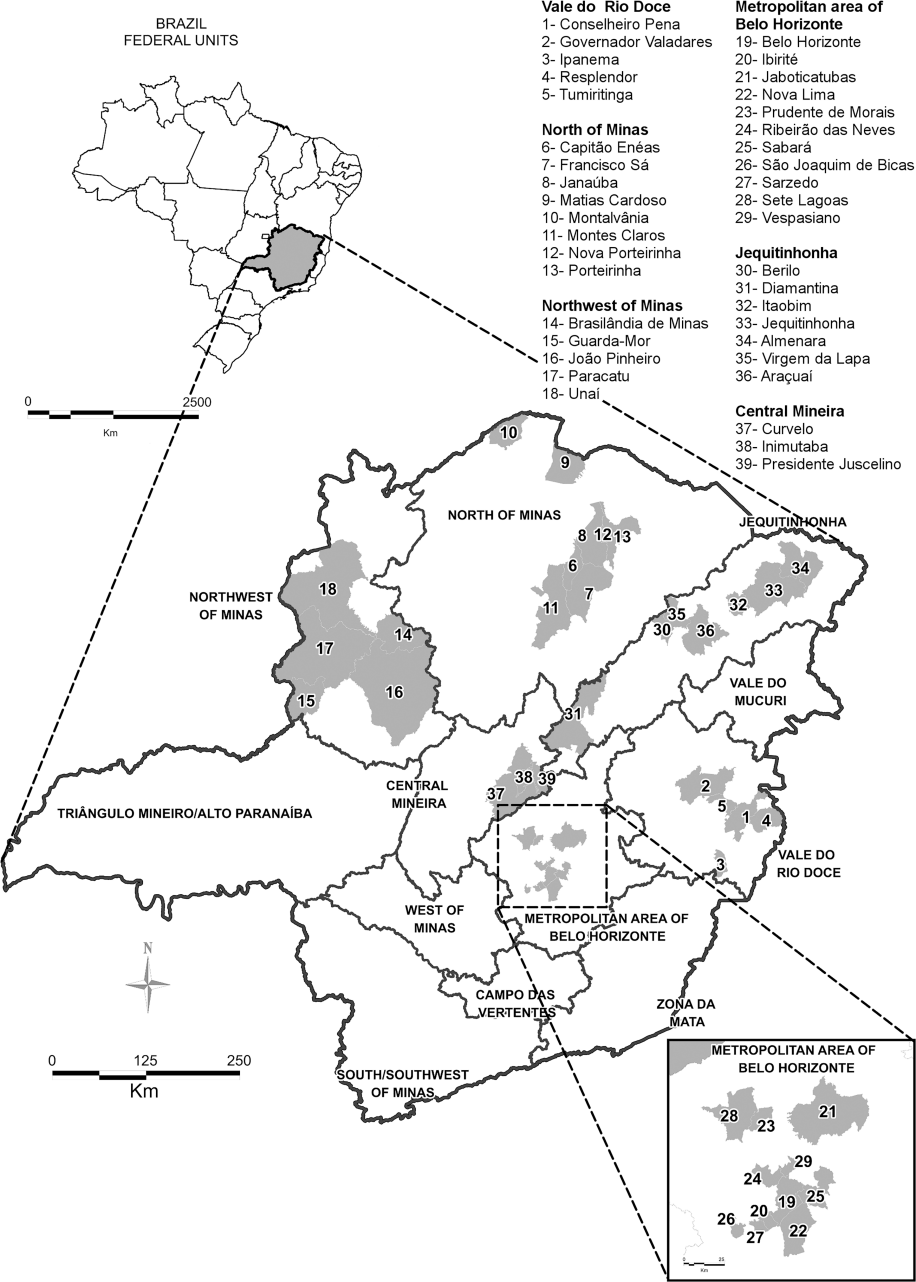
Mesoregions of Minas Gerais, Brazil. ^a^ Municipalities that were cited or discussed in this study are in grey.

The Brazilian Institute of Geography and Statistics (IBGE) divides Minas Gerais in 12 mesoregions (Fig 1): Campo das Vertentes; Central Mineira; Jequitinhonha; Metropolitan Area of Belo Horizonte; Northwest of Minas; North of Minas; West of Minas; South/Southwest of Minas; Triângulo Mineiro/Alto Paranaíba; Vale do Mucuri; Vale do Rio Doce; and Zona da Mata [36]. Each mesoregion is an aggregate of municipalities from the same geographic area presenting similar social and economic profiles and natural conditions.

### Source of data

In Brazil, VL is a disease of compulsory notification, i. e., in the case of clinically suspected VL, health professionals must fill in a specific SINAN/VL form to start up procedures to investigate the disease. Initially, data such as the home address of the patient, age, gender, schooling, occupation, date when the first symptoms erupted, date of notification, and clinical manifestations (signs and symptoms) are collected. Later, additional information such as laboratorial exam results, date of treatment commencement, medication used, and outcomes are added to the system.

During the period analyzed in this study (2002–2013), the SINAN/VL database changed platform from Windows-based version (the ILeishVi 2002–2006) to Net version (LEISHNET, 2007–2013). Therefore, the databases had to be standardized and a new unified dataset had to be generated with the same variables for analyzes using a Microsoft Office Excel 2013.

**Fig 2:**
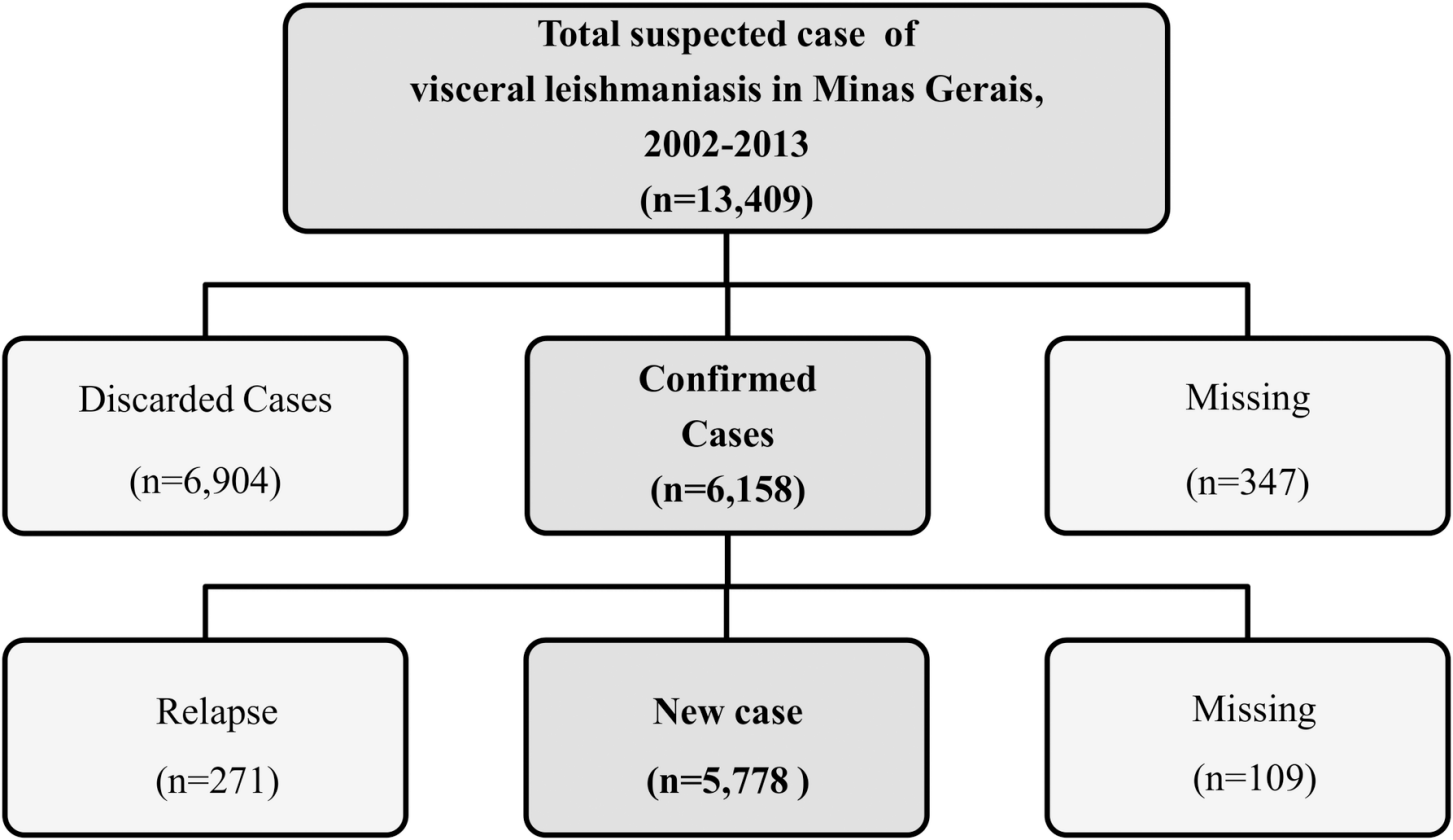
Fluxogram of the population analysed.

In the period analyzed herein, 13,409 suspected cases of VL were registered in the SINAN/VL. The number of confirmed cases was 6,158 (46%), 6,904 (51.5%) cases were discarded because the disease was not confirmed, and 347 (2.5%) cases had notification forms lacking information regarding the course of the disease. Among the confirmed cases, only new cases were included in the present study, totaling 5,778 cases (Fig 2).

### Statistical analysis

The data were analyzed in two phases. First, thematic maps of the state of Minas Gerais were generated with the crude and the smoothed cumulative incidence rates of VL for each municipality within the mesoregions of the state. The software *MapInfo 10*.*0* (MapInfo Corporation, Troy, New York) and *TerraView 4*.*2*.*2* (Instituto Nacional de Pesquisas Espaciais, INPE, SP, Brazil) were used. The information regarding the estimated resident population of each municipality and the cartographic basis of the state were obtained from IBGE [37].

Because the analyzes covered 12 years (2002–2013) of notifications, the data involved was divided into four maps, each comprising three years of study: 2002–2004, 2005–2007, 2008–2010 and 2011–2013. The intervals of incidence rates used were chosen using the software *MapInfo 10*.*0* considering the quartile, mean, median, and minimum and maximum values.

The cumulative crude incidence was calculated using Microsoft Office Excel 2013, and the smoothed cumulative incidence was obtained with the software *TerraView 4*.*2*.*2*. The local empiric Bayesian estimator, which allows for the estimation of the incidence of a municipality using the incidence rates of the neighboring municipalities converging to a local mean, was used. To evaluate the spatial variability of the data, a spatial proximity matrix using contiguity-based spatial weights was built. The elements of this matrix can take values 1 (if geographical analytical units are adjacent) or 0 (otherwise) [38]. Following the calculations, thematic maps for both the crude and the smoothed cumulative incidence rates were generated using *MapInfo 10*.*0*.

Next, Generalized Linear Models (GLM), through Poisson Regression, using STATA version 12.0 software (Stata Corp., College Station, TX, USA), was used to quantify the variation of the average number of VL cases from one year to the next in each of the 12 mesoregions of Minas Gerais. A curve with the annual incidence rates was generated for each mesoregion. Visual inspection of the resulting graphs allowed the establishment of cut off points according to the trends of increase or decrease of the number of VL cases over time. Consequently, the time interval analyzed was divided differently for each mesoregion.

The response (or dependent) variable was the “number of cases”, and the “year” was used as the independent variable in the temporal series. The logarithm of the population [log(pop)] was used as the “*Offset*” term, i. e., was a known component included for adjustment of the model. The equation used was as follows:

$$\mathrm{Log}\;\left(\mu_{i}\right)=\log\left({\mathrm{pop}}_{i}\right)+\alpha +\beta *\mathrm{year}$$

Where:

pop_i_ = Population of the year, with i = 1, 2, 3,…, 12, representing the years from 2002 to 2013.
μ_i_ = mean number of VL cases for year i, i = 1, 2, 3,…, 12.
α = value of the model’s constant
β = slope coefficient
year = 1, 2, 3,…, 12, representing the years from 2002 to 2013.

The equation (e^β^-1)*100% was used to obtain the variation of the mean number of VL cases from one year to the following.

## Results

### Visceral leishmaniasis cases

The present study only included new cases of VL, which totaled 5,778. Of these, 89% were confirmed by at least one diagnostic test (ELISA and/or IFAT and/or parasitological). Also, the SINAN /VL’s platform (LEISHNET, n = 3349) included one variable informed that 95% of the cases were considered positive by clinical-laboratory criteria, while only 5% were confirmed by the clinical-epidemiological criteria. Therefore, we consider that the cases studied were correctly classified as VL. Of the cases analyzed, 91% were from urban areas.

### Characterization of the mesoregions of Minas Gerais

The mesoregions of Central Mineira, Jequitinhonha, Metropolitan area of Belo Horizonte, Northwest of Minas, and North of Minas concentrated most VL cases (91%) and the highest number of municipalities with registered cases (66%). Among those, the Metropolitan area of Belo Horizonte mesoregion was the one with the largest population, the highest number, and the widest variation of VL cases, considering the population of its municipalities. On the other hand, the mesoregions of Campo das Vertentes, South/Southwest of Minas, Vale do Mucuri, and Zona da Mata presented the lowest number of cases (1%) and the lowest number of municipalities which reported VL cases (13%) (Table 1).

**Table 1:** Population, municipalities and occurrence of visceral leishmaniasis (VL) in the mesoregions of Minas Gerais, Brazil.

Mesoregion	Populational mean^a^ ±SD^b^	Population (Min-Max)^c^	Number of municipalities per mesoregion	Municipalities with VL cases n (%)	Number of VL cases
Campo das Vertentes	15176 ±26317	(2162–124253)	36	6 (16.7%)	9
Central Mineira	13518 ±16115	(863–73200)	30	18 (60.0%)	192
Jequitinhonha	13693 ±10299	(2979–45094)	51	35 (68.6%)	335
Metropolitan area of Belo Horizonte	59154 ±241504	(1628–2378246)	105	65 (61.9%)	2880
Northwest of Minas	18829 ±22954	(3276–83008)	19	13 (68.4%)	643
North of Minas	17887 ±37964	(2931–349098)	89	62 (69.7%)	1192
West of Minas	21062 ±33872	(1444–207352)	44	14 (31.8%)	57
South/Southwest of Minas	16593 ±23191	(1716–150594)	146	13 (8.9%)	18
Triângulo Mineiro/ Alto Paranaíba	31574 ±81476	(1425–592800)	66	17 (25. 8%)	46
Vale do Mucuri	16495 ±25683	(2798–130453)	23	4 (17.4%)	13
Vale do Rio Doce	15679 ±36122	(2386–259549)	102	27 (26.5%)	369
Zona da Mata	15178 ±44565	(1674–506610)	142	16 (11.3%)	24
**TOTAL**	**22718±93564**	**(863–2378246) **	**853**	**290 (34.0%)**	**5778**

^a^Mean of the population per mesoregion in the period between 2002 and 2013. All the municipalities within each mesoregion were considered.

^b^SD: Standard Deviation

^c^Minimum and maximum population per municipality among all the municipalities within the mesoregion.

### Spatial distribution–thematic maps of the crude and smoothed cumulative incidence rates of VL

**Fig 3:**
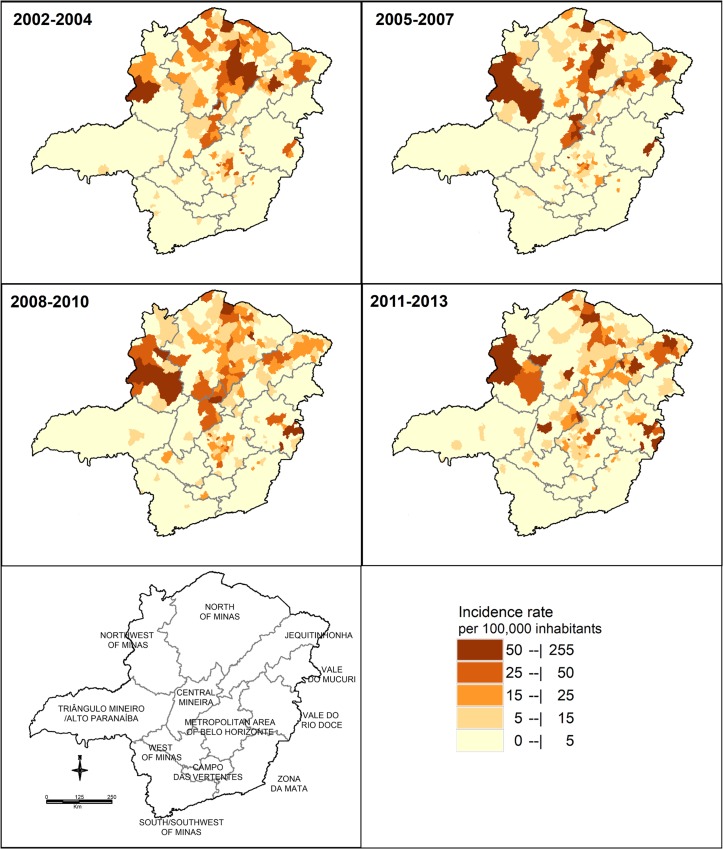
Thematic maps of the crude cumulative incidence of visceral leishmaniasis, 2002–2013, Minas Gerais, Brazil.

**Fig 4:**
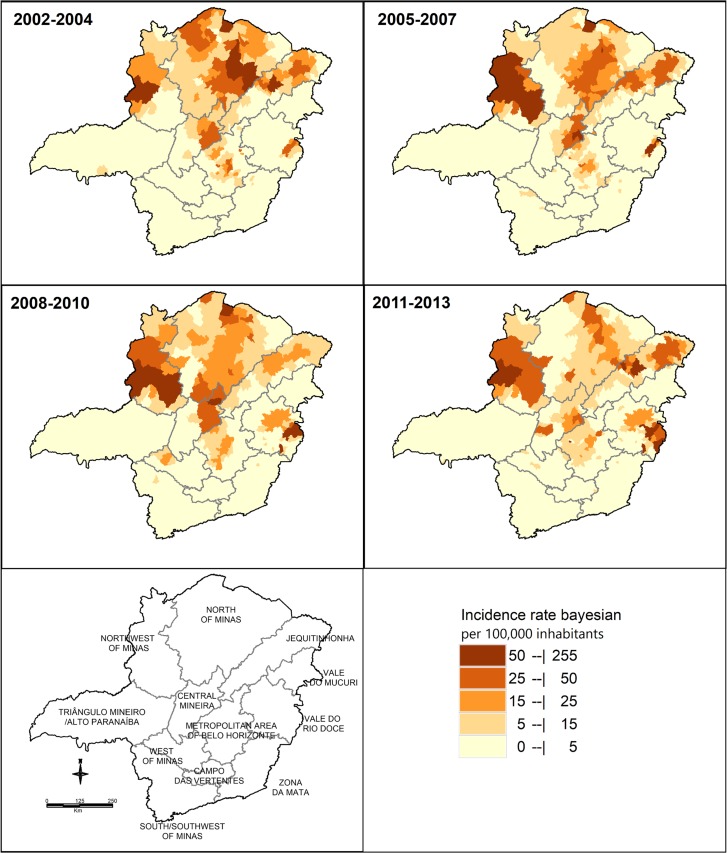
Thematic maps of the smoothed cumulative incidence of visceral leishmaniasis, 2002–2013, Minas Gerais, Brazil.

The variation of the incidence from 2002 to 2013 was obtained from the thematic maps of crude (Fig 3) and smoothed (Fig 4) cumulative incidence rates, for each municipality of the 12 mesoregions of Minas Gerais. The highest incidence rates concentrated in the mesoregions located in the north (Northwest of Minas, North of Minas, and Jequitinhonha), east (Vale do Rio Doce), and central (Central Mineira and Metropolitan area of Belo Horizonte) parts of the state (Table 2).

**Table 2:** Number of cases and incidence rates of visceral leishmaniasis per 100,000 inhabitants in each mesoregion of Minas Gerais, Brazil, 2002–2013.

MESOREGION	2002–2004		2005–2007		2008–2010		2011–2013	
	Cases	Incidence rate	Cases	Incidence rate	Cases	Incidence rate	Cases	Incidence rate
Campo das Vertentes	1	0.2	1	0.2	4	0.7	3	0.5
Central Mineira	46	11.8	54	13.32	49	11.8	43	10.3
Jequitinhonha	73	10.7	92	13.3	59	8.2	111	15.8
Metropolitan area of Belo Horizonte	655	11.1	777	12.3	909	14.0	539	8.5
Northwest of Minas	94	27.4	241	67.7	192	52.4	116	31.2
North of Minas	458	29.9	290	18.2	267	16.2	177	10.9
West of Minas	9	1.0	6	0.7	22	2.3	20	2.1
South/Southwest of Minas	8	0.3	5	0.2	4	0.2	1	0.0
Triângulo Mineiro/ Alto Paranaíba	6	0.3	7	0.3	18	0.8	15	0.7
Vale do Mucuri	1	0.3	3	0.8	4	1.0	5	1.3
Vale do Rio Doce	17	1.1	16	1.0	166	10.1	170	10.4
Zona da Mata	5	0.2	5	0.2	7	0.3	7	0.3

During the first three trienniums, the mesoregions of Northwest of Minas and North of Minas presented areas with the highest crude cumulative incidence rates. In the last triennium, however, a discrete decrease was observed in the rates in both these mesoregions. The highest incidence rate throughout the study, and among all the mesoregions, was observed in the Northwest of Minas mesoregion in the second triennium (67.7/100,000 inhabitants). This mesoregion presented a considerable increase in the incidence rates from the first to the second triennium and a subsequent reduction in the last two trienniums. Nonetheless, it was the mesoregion with the highest incidence rate within the state of Minas Gerais in the last triennium (31.2/100,000 inhabitants).

Paracatu, one of the most populous municipalities of the Northwest mesoregion, presented high VL incidence rates throughout the period of study (approximately 60/100,000 inhabitants). There was an increase in the incidence rates during the full study period in the municipalities of Unaí and Brasilândia de Minas, where VL cases were already observed between 2002 and 2004. Moreover, new cases arose in other municipalities (such as João Pinheiro and Guarda Mor), indicating the expansion of the disease in this mesoregion (Fig 3). This information is visualized in the map of smoothed incidence (Fig 4). In the last triennium (2011–2013), however, a slight reduction was observed in the number of municipalities that presented cases of VL (Figs 3 and 4).

The North of Minas mesoregion presented a high number of municipalities with VL cases during the four trienniums evaluated (17 municipalities). Among them, Montes Claros, Porteirinha, Matias Cardoso, Janaúba, Capitão Enéas, Montalvânia, Francisco Sá, and Nova Porteirinha showed the highest incidence rates. Figs 3 and 4 show the reduction in the incidence rates of VL in the municipalities of this mesoregion over the period analyzed. The incidence reduced from 29.9/100,000 inhabitants in the first triennium to 10.9/100,000 inhabitants in the last. This mesoregion showed the largest reduction in the incidence between the first and the second periods, as shown by the smoothed incidence rate maps (Fig 4).

The Vale do Rio Doce mesoregion had the largest increase in the incidence rates over the temporal series. Although incidence rates in this region remained stable during the first two trienniums (1.1 and 1.0/100,000 inhabitants, respectively), a considerable increase was observed in the last two (10.1 and 10.4/100,000 inhabitants, respectively). This increase reflected the expansion of VL to the east and the center of the mesoregion, particularly to the municipalities of Governador Valadares, Conselheiro Pena, Ipanema, Resplendor, and Tumiritinga (Figs 3 and 4).

The Central Mineira and Metropolitan area of Belo Horizonte mesoregions had small oscillations in the incidence rates of VL throughout the study period (Table 2). Nevertheless, the incidence rates reduced in the final triennium (10.3 and 8.5/100,000 inhabitants, respectively) in relation to the initial (11.8 and 11.1/ 100,000 inhabitants). The following municipalities showed incidence rates higher than 5/100,000 inhabitants in all four trienniums: Curvelo, Presidente Juscelino, and Inimutaba (all within the Central Mineira mesoregion); Belo Horizonte, Ribeirão das Neves, Sabará, Ibirité, Prudente de Morais, Vespasiano, Jaboticatubas, Sarzedo, Sete Lagoas, São Joaquim de Bicas, and Nova Lima (all within the Metropolitan area of Belo Horizonte mesoregion) (Figs 3 and 4).

Slight oscillations in VL incidence rates were also observed in the Jequitinhonha mesoregion. This oscillation is difficult to detect in the maps because different municipalities presented VL cases at various times (Figs 3 and 4). During all the time intervals evaluated, only seven municipalities (Berilo, Araçuaí, Jequitinhonha, Almenara, Itaobim, Diamantina, and Virgem da Lapa) stood out with very intense colors in the maps. In the last triennium (2011–2013) the incidence rate increased (15.8/100,000 inhabitants) in comparison with the three previous trienniums (10.7 for the first; 13.3 for the second, and 8.2/100,000 inhabitants for the third).

The other mesoregions (Campo das Vertentes, West of Minas, South/Soutwest of Minas, Triângulo Mineiro/Alto Paranaíba, Vale do Mucuri, and Zona da Mata) reported few VL cases and, consequently, low incidence rates throughout the study (Figs 3 and 4). Thus, these mesoregions were less important, as well as their variations over time in relation to VL.

### Generalized Linear Model–Poisson Regression

**Fig 5:**
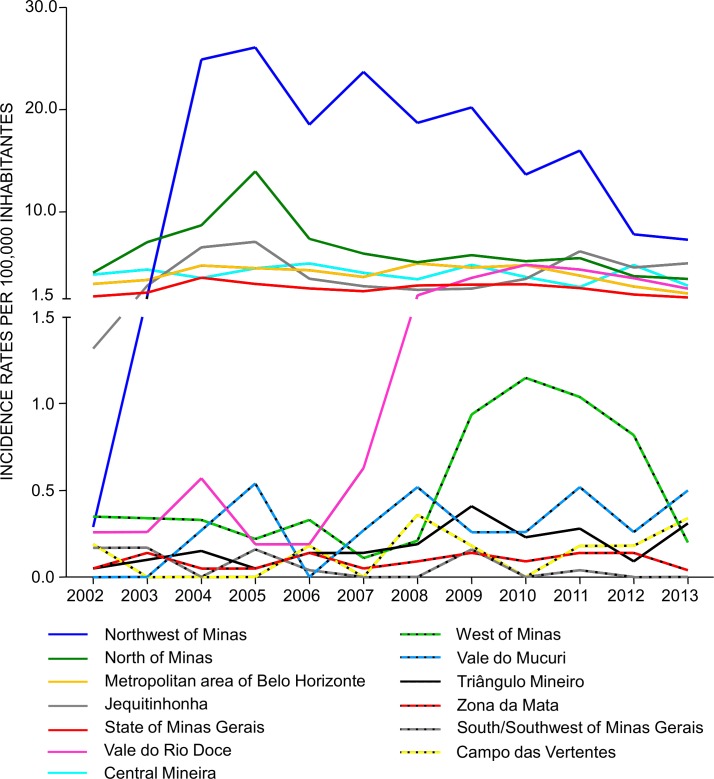
Incidence rates of visceral leishmaniasis in the mesoregions of Minas Gerais, 2002–2013.

Fig 5 shows the crude VL incidence rates obtained for each mesoregion between 2002 and 2013. These rates fluctuated over time and from one mesoregion to another. The following mesoregions reached elevated incidence rates, ranging from 0 to 26/100,000 inhabitants: Central Mineira, Jequitinhonha, Metropolitan area of Belo Horizonte, Northwest of Minas, North, of Minas and Vale do Rio Doce. On the other hand, the following mesoregions presented low VL incidence rates, reaching a maximum of 1.2/100,000 inhabitants: Campos das Vertentes, West of Minas, South/Southwest of Minas, Triângulo Mineiro/Alto Paranaíba, Vale do Mucuri, and Zona da Mata (Fig 5). The VL incidence rates in the whole state of Minas Gerais varied from 1.6 to 3.5/100,000 inhabitants.

The time periods used in the Poisson Regression model were selected by visual analysis of the graph presented in Fig 5. For each mesoregion, different time intervals were chosen, considering the intervals of increase or reduction in the incidence rates per mesoregion. The results of the adjustment of the model are shown in Table 3.

**Table 3:** Poisson Model, variation in the average number of visceral leishmaniasis cases per year in the mesoregions of Minas Gerais, Brazil, 2002–2013.

Mesoregion	Period	Coefficient (β)	Exponential of β CI^a^95%	%^b^	*p*
Campo das Vertentes	2002–2013	0.13	1.14 (0.93; 1.40)		0.197
Central Mineira	2002–2013	0.01	0.98 (0.94; 1.02)		0.395
Jequitinhonha	2002–2005	0.51	1.66 (1.39; 1.98)	66	0.000^c^
	2005–2008	-0.39	0.68 (0.56; 0.80)	-32	0.000 ^c^
	2008–2013	0.16	1.17 (1.07; 1.28)	17	0.001 ^c^
Metropolitan area of Belo Horizonte	2002–2008	0.05	1.05 (1.03; 1.08)	5	0.000 ^c^
	2008–2013	-0.16	0.85 (0.82; 0.88)	-15	0.000 ^c^
Northwest of Minas	2002–2005	0.92	2.52 (2.11; 3.00)	152	0.000 ^c^
	2005–2013	-0.12	0.88 (0.85; 0.91)	-12	0.000 ^c^
North of Minas	2002–2004	0.36	1.43 (1.27; 1.61)	43	0.000 ^c^
	2004–2013	-0.13	0.87 (0.85; 0.89)	-13	0.000 ^c^
West of Minas	2002–2007	-0.14	0.87 (0.64; 1.17)		0.357
	2007–2010	0.73	2.08 (1.32; 3.26)	108	0.002 ^c^
	2010–2013	-0.41	0.66 (0.48; 0.93)	-34	0.017 ^c^
South/Southwest of Minas	2002–2013	-0.22	0.79 (0.68; 0.94)	-21	0.005 ^c^
Triângulo Mineiro/Alto Paranaíba	2002–2009	0.25	1.28 (1.06; 1.55)	28	0.009 ^c^
	2009–2013	-0.13	0.88 (0.68; 1.14)		0.324
Vale do Mucuri	2002–2013	0.12	1.13 (0.95; 1.33)		0.162
Vale do Rio Doce	2002–2006	-0.07	0.93 (0.70; 1.24)		0.628
	2006–2010	0.64	1.90 (1.67; 2.17)	90	0.000 ^c^
	2010–2013	-0.21	0.81 (0.72; 0.91)	-19	0.000 ^c^
Zona da Mata	2002–2013	0.03	1.03 (0.92; 1.16)		0.629

^a^CI: Confidence Index

^b^Annual percentage variation in the average number of VL cases, i. e., [(exp(β) -1]*100%

^c^Significant p values (α = 0.05)

A single period (2002–2013) was chosen to analyze the mesoregions of Campos das Vertentes, Central Mineira, South/Southwest of Minas, Vale do Mucuri, and Zona da Mata). This was because it was not possible to observe trends in growth or reduction in the average number of VL cases occurring in these mesoregions, given the instability in the number of cases observed in the temporal series analyzed (Fig 5). The results obtained with the Poisson regression corroborated the analysis of the graph, as the results of the model were not significant, except in the case of the mesoregion South/Southwest of Minas. Indeed, the variation in the average number of VL cases per year was relatively stable during the study period (Table 3). However, the slope coefficient (β) obtained for the South/Southwest of Minas mesoregion was -0.22, indicating a reduction in the average number of cases between the beginning and the end of the period analyzed, despite the fluctuations observed over the years (Fig 5).

The Metropolitan area of Belo Horizonte, Northwest of Minas and North of Minas mesoregions presented similar results. Table 3 showed that, in the initial periods, the average number of cases per year rose 5%, 152%, and 43%, respectively. In the last periods, the average number of cases reduced in 15%, 12% and 13%, respectively. Of note is the mesoregion Northwest of Minas, which exhibited a marked increase in the average number of VL cases between the years of 2002 and 2005 (152%).

The time intervals analyzed in the West of Minas and Vale do Rio Doce mesoregions were divided into three periods. It was not possible to establish a significant increase or reduction in the average number of cases per year, in the first periods analyzed (Table 3). In the intermediate intervals, the coefficients were positive, demonstrating an increase in the average number of cases (108% in the West of Minas and 90% in Vale do Rio Doce). On the other hand, the last periods showed a reduction of 34% (West of Minas) and of 19% (Vale do Rio Doce) in the number of VL cases from one year to the next.

Two periods were chosen to analyze the Triângulo Mineiro/Alto Paranaíba mesoregion: 2002–2009 and 2009–2013. In the first period, the mesoregion presented a positive slope coefficient of the model (0.25), with an increase of approximately 28% in the average number of VL cases from one year to the next. In the interval between 2009 to 2013, the variations were not significant and it was not possible to establish a co-relation between the average number of cases from one year to the next. Thus, the average number of cases remained relatively constant during this period (Table 3).

Three periods were chosen to analyze the Jequitinhonha mesoregion: 2002–2005, 2005–2008, and 2008–2013. In the first interval, the model coefficient was 0.51, indicating an increase of approximately 66% in the average number of cases from one year to the next. On the other hand, the coefficient was negative (-0.39) in the period between 2005 and 2008, demonstrating a reduction of 32% in the average number of cases. During the final period, this mesoregion was the only one that presented a positive coefficient (0.16), with the average number of cases increasing by 17% from one year to the next.

## Discussion

The present study shows that VL had a heterogeneous spatial and temporal distribution in the state of Minas Gerais, in the period between 2002 and 2013. Among the 12 existing mesoregions, six (Central Mineira, Jequitinhonha, Metropolitan area of Belo Horizonte, Northwest of Minas, North of Minas, and Vale do Rio Doce) were responsible for the expansion and maintenance of VL in the state. Among them, the Vale do Rio Doce and Jequitinhonha mesoregions presented a considerable increase in the incidence rates of the disease in the last triennium (2011–2013). The North of Minas Gerais and Metropolitan area of Belo Horizonte mesoregions reduced the incidence rates in the last years of the study, despite the elevated number of VL cases. In the other six mesoregions (Campo das Vertentes, West of Minas, South/Southwest of Minas, Triângulo Mineiro/Alto Paranaíba, Vale do Mucuri, and Zona da Mata), only sporadic cases of the disease were reported during the study period and, consequently, these regions showed low and unsteady VL incidence rates.

VL is expanding in Brazil [2] and in other South American countries [39]. In Argentina [40,41] and Paraguay [42,43] a significant increase in the number of VL cases was observed in the last two decades, raising concerns about the spreading of the disease. Therefore, studies evaluating the process of expansion of VL and the spatial and temporal variation of its incidence are of great importance.

The Metropolitan area of Belo Horizonte mesoregion comprises a large number of municipalities (105) and stands out from the other mesoregions of Minas Gerais for being the most urbanized and the most economically developed, and where the political, financial, commercial, educational, and cultural centers of the state are concentrated [36]. Dissemination and urbanization of VL became even more evident in the Metropolitan region of Belo Horizonte after an autochthonous case of VL was registered in the municipality of Sabará [10]. The results presented herein reveals that since 2002 this municipality has higher, albeit oscillating, VL incidence rates than other municipalities of the same mesoregion considered of high VL transmission risk such as Sarzedo, Jaboticatubas, Sete Lagoas, and Vespasiano. Indeed, the 1990’s saw increasing numbers of VL cases being registered in the Metropolitan area of Belo Horizonte mesoregion and that trend persisted until the early 2000’s [44]. Previous studies in this region pointed out that the VL cases occurred in non-rural areas, reinforcing the urbanization process of the disease [44], which becomes more likely in localities of high population density (Table 1) and with houses close to one another [45]. The present study suggests that this trend persisted at least up to 2008 since map analysis detected a decreasing trend only in the last years analyzed. Indeed, the Poisson regression adjustment indicated an increase in the average number of cases between the years of 2002 and 2008 (5%) and a reduction between 2008 and 2013 (-15%).

Recent studies have shown that VL cases are heterogeneous in the capital Belo Horizonte [16,46]. This may be due to the city’s vast territorial extension, high population density, and different microenvironments [47]. The VLCSP implemented in Belo Horizonte in the 1990’s [48] is considered to take place in a systematic and orderly manner throughout the city [49] and this may explain the reduction in the VL incidence rates observed in the present study. It is possible that the situation of Belo Horizonte reflects in the adjacent municipalities as well as in the mesoregion of the Metropolitan area of Belo Horizonte. This may be due to the population densification in these areas [45,47], which compromises the execution and maintenance of measures to control the disease. Therefore, both Belo Horizonte and its mesoregion presented similar incidence rates, with a gradual increase in the incidence of VL in the first trienniums and a decrease in the last.

Different from the Metropolitan area of Belo Horizonte mesoregion, the North of Minas mesoregion presents more ancient cases of the disease [8]. Nevertheless, research performed in some municipalities of this mesoregion, such as Montes Claros [8,50] and Porteirinha [51] pointed out that the incidence of VL is decreasing in the last years. The authors attributed this decrease to the VL control actions that are being performed in these municipalities. This reduction in the number of cases in this mesoregion is in agreement with the results obtained herein. Accordingly, the results of the Poisson regression adjustment, revealed a reduction in the average number of VL cases in the North of Minas from 2004 onwards (-13%) and the thematic maps, presented similar data to those previously described in Montes Claros in the same period [8]. Indeed, while investigating the VL cases registered in Montes Claros between 2001 and 2007, these authors observed that, from 2005 onwards, the incidence of VL reduced, but the municipality remained endemic for the disease and representing a serious public health problem [8]. Because VL is associated with poor socioeconomic conditions [2,12,31] and that Montes Claros is considered the municipality with the best socioeconomic indexes in this mesoregion, one can assume that the problem regarding VL extends to the whole of the North of Minas mesoregion.

The thematic maps indicated that the Northwest of Minas and Vale do Rio Doce mesoregions had pronounced increase of VL cases during the study period. On the other hand, the Poisson regression showed that, despite the increase observed in some periods, there was a reduction in the incidence rates starting in 2005 in the Northwest of Minas and in 2010 in the Vale do Rio Doce. In 2005, VL expanded to other municipalities in the Northwest of Minas mesoregion. Initially, Unaí and Paracatu stood out as typical examples of VL urbanization [14]. Later, neighboring municipalities, such as João Pinheiro and Brasilândia de Minas, also began to show a high incidence of the disease. Noteworthy, the four mentioned municipalities occupy a vast territorial extension in the mesoregion and kept a cumulative incidence superior to 20/100,000 inhabitants in the last years. The high incidence of VL in the first three periods analyzed in the thematic maps and its reduction during the last period in the mesoregions North of Minas and Northwest of Minas may be explained by their geographical proximity.

The thematic maps generated for the Vale do Rio Doce mesoregion for the period between 2002 and 2004 reveal only five municipalities with cumulative incidence higher than 5/100,000 inhabitants. From 2008 onwards, expansion of VL is observed in this mesoregion with 10 municipalities presenting VL incidence rates higher than 5/100,000 inhabitants, in which stands out Governador Valadares. In this mesoregion, human VL cases started to be reported the 1960’s [9]. At that time, control measures such as the treatment of VL patients with Glucantime, elimination of dogs positive for VL, and use of the insecticidal dichlorodiphenyltrichloroethane–DDT in the domicile and peridomicile areas were established in the municipalities with an expressive number of cases. These measures resulted in a progressive reduction in the number of cases and the absence of VL in the years of 1978 and 1979 [52]. As an unfortunate consequence, disease control measures were interrupted in the 1990’s [18,19]. Since 2008, studies have identified cases of the disease in Governador Valadares [19,20], which may explain the increase in the incidence of VL in the mesoregion of Vale do Rio Doce in the same year and the increase in the average number of cases between 2006 and 2010. The urbanization process that is taking place in the municipality of Governador Valadares and the interruption of the disease control measures previously implemented in this mesoregion [19] may be the reasons underlying the observed increase in the number of cases VL. These results indicate the relevant role of Governador Valadares on the increase in the number of cases of the Vale do Rio Doce mesoregion in the last years analyzed.

The results presented herein showed the geographical expansion of VL in Minas Gerais between 2002 and 2013. The data reveal a trend for VL persisting in municipalities that already presented cases, even when there was oscillation in the disease incidence rates. Despite this expansion, the incidence of the disease does not spread from the North region of the state to the South, that is, to mesoregions such as Campos das Vertentes and South/Southwest of Minas, which presented a consistently low number of cases.

The persistence of the disease in the mesoregions located in the North of Minas Gerais may be related to socioeconomic [5,6,31] and environmental [6] factors. A study performed in Montes Claros showed that VL in this region is associated with poor domiciles and precarious sanitation conditions, which facilitates the accumulation of organic material and other factors that enable the life cycle of the main vector of VL, *Lutzomya longipalpis* [12]. The maintenance of the disease cycle explained by similar reasons was also observed in the Jequitinhonha (Araçuaí) [53]. The findings reported herein corroborate these previous observations, since the highest VL incidence rates were found in the North of Minas Gerais, a region that presents low Human Development Index [54]. Considering the environmental factors, several authors described what Sherlock (1996) [55] previously observed in the northeastern state of Bahia: that VL is occurring with higher frequencies in hot dry climates common to the northern areas of the Minas Gerais state [12,56,57]. These observations are in agreement with the results presented in the current study since it detected high incidence rates especially in the mesoregions located at the north of the state, which also have a hot and dry climate.

We suggest that VL presence in the northern region of Minas Gerais is mainly due to two reasons: climate and socioeconomic factors. Indeed, we observed that VL is distributed in contiguous mesoregions, namely Northwest of Minas, North of Minas, and Jequitinhonha, all of which have low socioeconomic development and display a warm climate favorable to the development of the vector [1]. On the other hand, the southern mesoregions, namely West of Minas, Campo das Vertentes, Zona da Mata, and South / Southwest of Minas, present lower temperatures and better socioeconomic conditions. Interestingly, although the mesoregion Triangulo Mineiro / Alto Paranaíba has a hot climate, it also presents few VL cases. This mesoregion is the second largest economy in the state and has the highest Human Development Index. The Human Development Index of the Belo Horizonte Metropolitan mesoregion is one the highest in the state of Minas Gerais, but there is a great socioeconomic disparity between the municipalities composing this mesoregion. The high levels of social inequality, as represented by the existence of slums within the municipality of Belo Horizonte itself, may underlie the high number of VL cases observed in this city.

This study has a few limitations that should be pointed out. Even though VL is of compulsory notification in Brazil, the real number of cases may be underestimated as the symptoms of the disease are unspecific, and some cases may go unreported. This might compromise calculations of the incidence and the estimates obtained through the Poisson regression. However, the extent of this underreporting is most probably minimum since SINAN covers all health systems (public and private) at its various levels of complexity. Furthermore, it is noteworthy that the medication used for treatment is solely dispensed by the Brazilian Health Public System and this measure has minimized underreporting.

Some variables of this system, such as “Final Classification” (cases that were confirmed, discarded or had incomplete forms) and “Type of entry” (New Case, Recurrence, Transference, and Ignored) are not filled after closure of the case, thus, increasing the number of losses (*missing*) and compromise the analyzes. Lastly, areas bordering the state of Minas Gerais were not evaluated. Furthermore, an empiric local Bayesian approach was used in one of the sets of the thematic maps created. This method estimates the mean local incidence in each municipality taking into account the incidence values of the neighboring municipalities. Thus, the rates generated are corrected, smoothed, and less unstable. Maps built using this approach are, therefore, more informative and interpretative.

The spatial and temporal epidemiology, together with the Poisson regression approach, allowed a more precise identification and quantification of areas of expansion and stabilization of VL, and the identification of regions that share similar spatial patterns. The findings reported herein may help to guide the implementation of actions for controlling the disease in the state of Minas Gerais. Given the worrisome expansion of VL in Brazil and in other South American countries, the results describing the spatial and temporal pattern of VL expansion in this vast geographic area may be relevant to researchers following the disease in other regions of Brazil and the world.

## Supporting information

S1 Checklist STROBE checklist. (DOC)
