# Supplementary material for: Spatial and temporal trends of visceral leishmaniasis by mesoregion in a southeastern state of Brazil, 2002-2013
Source: PLoS Negl Trop Dis. 2017 Oct 6;11(10):e0005950. doi: 10.1371/journal.pntd.0005950 (PMC5646873; doi:10.1371/journal.pntd.0005950)
Supplement: S1 Checklist — (DOC) [file pntd.0005950.s001.doc]

STROBE Statement—checklist of items that should be included in reports of observational studies

|  | Item No | Recommendation |
| --- | --- | --- |
| **Title and abstract** | 1 | (*a*) Indicate the study’s design with a commonly used term in the title or the abstract  Yes, included in the abstract |
|  |  | (*b*) Provide in the abstract an informative and balanced summary of what was done and what was found Yes |
| Introduction | | |
| Background/rationale | 2 | Explain the scientific background and rationale for the investigation being reported Yes |
| Objectives | 3 | State specific objectives, including any prespecified hypotheses Yes |
| Methods | | |
| Study design | 4 | Present key elements of study design early in the paper Yes |
| Setting | 5 | Describe the setting, locations, and relevant dates, including periods of recruitment, exposure, follow-up, and data collection Yes |
| Participants | 6 | (*a*) *Cohort study*—Give the eligibility criteria, and the sources and methods of selection of participants. Describe methods of follow-up Not applicable  *Case-control study*—Give the eligibility criteria, and the sources and methods of case ascertainment and control selection. Give the rationale for the choice of cases and controls Not applicable  *Cross-sectional study*—Give the eligibility criteria, and the sources and methods of selection of participants Not applicable |
|  |  | (*b*) *Cohort study*—For matched studies, give matching criteria and number of exposed and unexposed Not applicable  *Case-control study*—For matched studies, give matching criteria and the number of controls per case Not applicable |
| Variables | 7 | Clearly define all outcomes, exposures, predictors, potential confounders, and effect modifiers. Give diagnostic criteria, if applicable Yes |
| Data sources/ measurement | 8* | For each variable of interest, give sources of data and details of methods of assessment (measurement). Describe comparability of assessment methods if there is more than one group Yes |
| Bias | 9 | Describe any efforts to address potential sources of bias Yes |
| Study size | 10 | Explain how the study size was arrived at Yes |
| Quantitative variables | 11 | Explain how quantitative variables were handled in the analyses. If applicable, describe which groupings were chosen and why Yes |
| Statistical methods | 12 | (*a*) Describe all statistical methods, including those used to control for confounding Yes |
|  |  | (*b*) Describe any methods used to examine subgroups and interactions Yes |
|  |  | (*c*) Explain how missing data were addressed Yes |
|  |  | (*d*) *Cohort study*—If applicable, explain how loss to follow-up was addressed Not applicable  *Case-control study*—If applicable, explain how matching of cases and controls was addressed Not applicable  *Cross-sectional study*—If applicable, describe analytical methods taking account of sampling strategy Not applicable |
|  |  | (*e*) Describe any sensitivity analyses No |

Continued on next page

| Results | | |
| --- | --- | --- |
| Participants | 13* | (a) Report numbers of individuals at each stage of study—eg numbers potentially eligible, examined for eligibility, confirmed eligible, included in the study, completing follow-up, and analysed Yes |
|  |  | (b) Give reasons for non-participation at each stage Yes |
|  |  | (c) Consider use of a flow diagram Yes |
| Descriptive data | 14* | (a) Give characteristics of study participants (eg demographic, clinical, social) and information on exposures and potential confounders Not applicable - ecological study |
|  |  | (b) Indicate number of participants with missing data for each variable of interest Not applicable - ecological study |
|  |  | (c) *Cohort study*—Summarise follow-up time (eg, average and total amount) Not applicable |
| Outcome data | 15* | *Cohort study*—Report numbers of outcome events or summary measures over time Not applicable |
|  |  | *Case-control study—*Report numbers in each exposure category, or summary measures of exposure Not applicable |
|  |  | *Cross-sectional study—*Report numbers of outcome events or summary measures Not applicable |
| Main results | 16 | (a) Give unadjusted estimates and, if applicable, confounder-adjusted estimates and their precision (eg, 95% confidence interval). Make clear which confounders were adjusted for and why they were included Yes for the estimates obtained in the Generalized Linear Model (Poisson Regression). |
|  |  | (*b*) Report category boundaries when continuous variables were categorized |
|  |  | (*c*) If relevant, consider translating estimates of relative risk into absolute risk for a meaningful time period |
| Other analyses | 17 | Report other analyses done—eg analyses of subgroups and interactions, and sensitivity analyses Not applicable |
| Discussion | | |
| Key results | 18 | Summarise key results with reference to study objectives Yes |
| Limitations | 19 | Discuss limitations of the study, taking into account sources of potential bias or imprecision. Discuss both direction and magnitude of any potential bias Yes |
| Interpretation | 20 | Give a cautious overall interpretation of results considering objectives, limitations, multiplicity of analyses, results from similar studies, and other relevant evidence Yes |
| Generalisability | 21 | Discuss the generalisability (external validity) of the study results Yes |
| Other information | | |
| Funding | 22 | Give the source of funding and the role of the funders for the present study and, if applicable, for the original study on which the present article is based Yes |

*Give information separately for cases and controls in case-control studies and, if applicable, for exposed and unexposed groups in cohort and cross-sectional studies.

**Note:** An Explanation and Elaboration article discusses each checklist item and gives methodological background and published examples of transparent reporting. The STROBE checklist is best used in conjunction with this article (freely available on the Web sites of PLoS Medicine at http://www.plosmedicine.org/, Annals of Internal Medicine at http://www.annals.org/, and Epidemiology at http://www.epidem.com/). Information on the STROBE Initiative is available at www.strobe-statement.org.
